# Ectopic craniopharyngioma recurrence: a case report and literature review

**DOI:** 10.3389/fonc.2026.1880297

**Published:** 2026-07-08

**Authors:** Jiarun Tian, Hongliang Zhang, Yingjie Ding, Canghao Gao, Jiaming Liu, Tiantian Guo, Huanting Li

**Affiliations:** 1 Affiliated Hospital of Qingdao University, Department of Neurosurgery, Qingdao, China; 2 Qingdao West Coast New Area Central Hospital, Department of Neurosurgery, Qingdao, China

**Keywords:** adamantinomatous type, craniopharyngioma, ectopic recurrence, surgical tract implantation, temporal lobe

## Abstract

We reported a case of ectopic recurrence of craniopharyngioma (CP) in the right temporal lobe and review the relevant literature to investigate its clinical features, pathogenesis, diagnosis and management. A 67-year-old male patient presented with blurred vision and was diagnosed with adamantinomatous CP(aCP) in the sellar region. Four years postoperatively, he developed implantation metastasis in the right temporal lobe along the surgical tract, and achieved a favorable prognosis after complete lesion resection via reoperation. Based on literature review, ectopic recurrence of CP is mainly associated with tumor cell dissemination and implantation along the surgical tract or cerebrospinal fluid spread. The adamantinomatous variant is the predominant pathological subtype.Standardized intraoperative tumor-free techniques and maximal resection of the primary lesion are the core preventive strategies, and long-term regular follow-up is mandatory for postoperative patients.

## Introduction

Craniopharyngioma (CP) is a WHO grade I benign tumor, which is more common in the sellar area and suprasellar region. It originates from residual squamous epithelium of the CP during embryonic development. Although tumors are benign, they often cause significant endocrine and neurological symptoms due to their deep location and proximity to important neural structures. It accounts for 1%~3% of adult intracranial tumors and 5%~10% of children’s intracranial tumors, with an annual incidence rate of 0.13-2/100000. There is no significant gender difference, and there are two peak onset ages: the peak onset age for children is 5–14 years old, and the peak onset age for adults is 50–74 years old (1).

Adamantinomatous type CP (aCP) accounts for about 90%, and can occur in both children and adults; Squamous papillary type CP (pCP) accounts for about 10% and is more common in adults (2). The former is mostly cystic or cystic solid, prone to calcification, and often surrounds blood vessels. The latter is mostly solid or cystic, with rare calcification and no vascular wrapping (3). The aCP is characterized by high expression of β-catenin with typical abnormal nuclear accumulation. It also widely expresses squamous epithelial markers including CK5/6, p63 and CKpan, and exhibits higher positive rates of inflammatory factors and epithelial-mesenchymal transition-related proteins, with low to moderate Ki-67 proliferation index (4). In contrast, the pCP rarely presents ectopic nuclear β-catenin expression and mainly shows weak membranous and cytoplasmic expression. p63 expression is focal and weakly positive in pCP, while Claudin-1 and BRAF V600E mutation-associated proteins serve as its specific biomarkers (5).

The clinical manifestations are mainly pituitary hypothalamic dysfunction, visual impairment, and increased intracranial pressure (6).

In this article, we report on a 67-year-old male with ectopic recurrent CP and a review of the literature.

## Case presentation

A 67-year-old male patient was admitted to our hospital with a 6-month history of blurred vision. Cranial magnetic resonance imaging (MRI) revealed an oval abnormal signal lesion in the suprasellar region with a maximum diameter of approximately 26 mm. The lesion presented heterogeneous hypointensity on T1-weighted images and hyperintensity on T2-weighted and fluid-attenuated inversion recovery (FLAIR) sequences, causing compression of the optic chiasm. Honeycomb-like enhancement was observed after contrast administration (**Figure 1**).

**Figure 1:**
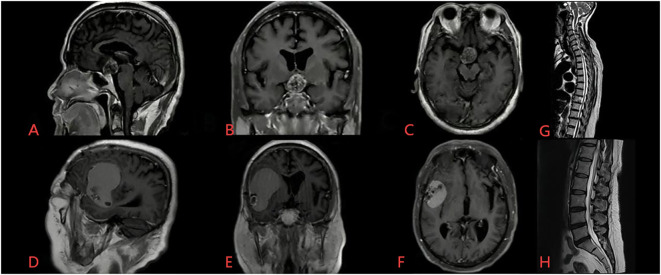
Preoperative MRI of the patient before two operations. **(A–C)** Images acquired prior to the first surgery; **(D–F)** Images acquired prior to the second surgery. **(A, D)** Sagittal view; **(B, E)** Coronal view; **(C, F)** Axial view. **(G, H)** Preoperative spinal cord MRI for the second surgery: No distant tumor metastasis was observed.

The patient underwent tumor resection via a right pterional approach. The sylvian fissure was routinely dissected, and the internal carotid artery cistern and chiasmatic cistern were opened along the anatomical space of the sylvian fissure to access the sellar region. The tumor was cystic-solid in composition. During dissection from the surrounding tissues, partial rupture of the tumor cyst wall occurred with a small amount of cyst fluid leakage. The cyst fluid was pale yellow, turbid and viscous. The surgical field was thoroughly irrigated with normal saline after tumor resection. Postoperative histopathological examination confirmed a diagnosis of aCP (**Figure 2**) Cranial computed tomography (CT) performed 1 month after the initial surgery confirmed gross total resection (GTR) of the tumor (**Figure 3**). The patient failed to attend regular follow-up examinations thereafter.

**Figure 2:**
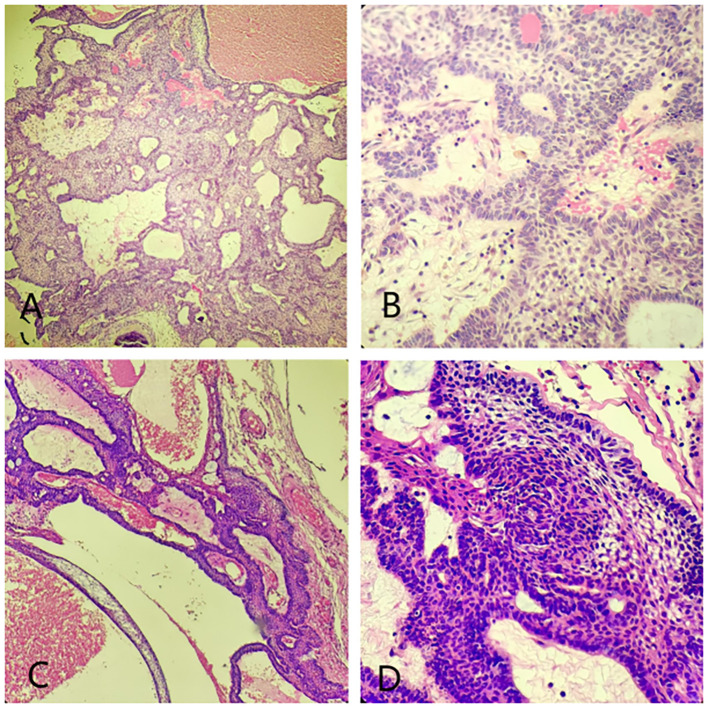
**(A, B)** are H&E-stained photos of primary craniopharyngioma and **(C, D)** are H&E-stained photomicrographs of ectopic recurrent craniopharyngioma. **(A, C)** H&E at 100×, **(B, D)** H&E at 400×.

**Figure 3:**
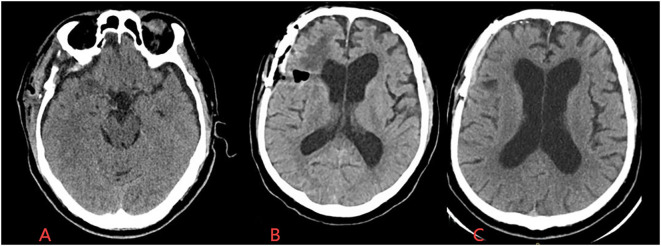
Postoperative CT images. **(A)** Follow-up CT at 1 month after the second surgery, showing total resection of the sellar tumor. **(B)** Follow-up CT at 1 year after the second surgery. **(C)** Follow-up CT at 2 years after the second surgery.

Four years after the initial surgery, the patient was readmitted to hospital due to headache. A full craniospinal magnetic resonance imaging workup, including complete cranial sequences and whole-spine MRI, was performed (**Figure 1**). The results revealed an isolated short T1 signal lesion in the right temporal lobe, with a maximum cross-sectional dimension of 50 mm × 49 mm, and no remarkable enhancement on contrast-enhanced scans (**Figure 1**). A brain abscess was initially suspected; nevertheless, typical ring enhancement was not observed on imaging, and routine blood tests were within normal ranges. Combined with the patient’s previous surgical history, the lesion was diagnosed as ectopic recurrent CP caused by tumor implantation along the original surgical tract. Intraoperatively, the recurrent lesion was found to have a large cystic component and presented an overall cystic-solid appearance.

The reoperation was performed via a right pterional approach. After dural opening, a cystic-solid tumor with high tension was exposed. The cyst wall was incised to release pale yellow, turbid, and viscous cyst fluid, which relieved the tumor tension. The cyst wall was then dissected free from the surrounding brain tissue, and the tumor was completely resected (**Figure 4**). After tumor resection, thorough hemostasis was achieved. The surgical field was repeatedly irrigated with dexamethasone solution and normal saline.Postoperative pathological examination confirmed the diagnosis of aCP again (**Figure 2**). Immunohistochemical staining revealed CKpan (+), CK5/6 (+), p40(+), β-catenin (membrane and nuclear positive), CK7 (focal positive), EMA (focal positive), Ki-67 (positive, 8%), GATA3 (−), GFAP (−) and p63 (+).The patient was followed up for 2 years. Cranial CT scans obtained at 1 year and 2 years after reoperation showed no signs of tumor recurrence (**Figure 3**).

**Figure 4:**
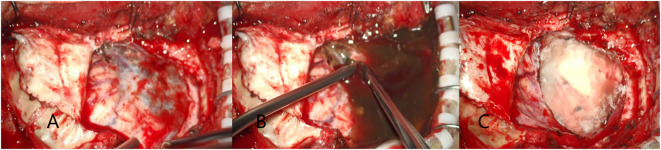
Intraoperative views during reoperation. **(A)** After dural incision, a cystic-solid tumor was visualized; **(B)** After incising the cyst wall, glistening yellowish-brown cyst fluid spurted out; **(C)** The cyst wall was dissected free from surrounding brain tissue, and gross total resection of the tumor was achieved.

## Discussion

Ectopic recurrent CP is very rare. We reviewed 72 patients with recurrent CP in the literature [**Supplementary Table 1** (7–62)].

Recurrent CP can occur at any age without significant sex predilection. The aCP is the predominant pathological subtype of recurrent CP, whereas pCP is extremely rare. The aCP is mainly composed of cystic or solid components, with a fragile tumor cyst wall that is prone to rupture. Once the cyst fluid overflows, tumor cells can easily spread and implant along the surgical tract or subarachnoid space, which serves as the key pathological basis for ectopic recurrence (7). In contrast, the pCP variant is predominantly solid, with lower invasiveness and a lower tendency to rupture, resulting in a significantly lower risk of ectopic recurrence.

In this case, the primary tumor had minimal cystic components and low cyst wall tension, while the recurrent lesion displayed extensive cystic changes with markedly elevated cyst wall tension. Cyst rupture occurred during the primary surgery. Despite thorough irrigation with a large volume of normal saline intraoperatively, residual tumor tissue remained. This led to tumor cell seeding along the surgical corridor and via cerebrospinal fluid circulation, which eventually resulted in ectopic tumor recurrence.

To prevent such ectopic recurrence, strict tumor-free operative principles must be followed during the initial surgery to avoid tumor rupture and achieve maximal resection of the primary lesion. If tumor rupture occurs intraoperatively, the surgical field and the entire surgical pathway should be thoroughly irrigated with normal saline, and residual cystic fluid as well as tumor fragments must be completely aspirated and cleared (8).

Gross total resection remains the first-line treatment for reducing the recurrence risk of CP (8). For lesions closely adhered to adjacent vital neurovascular structures, subtotal resection is a feasible option, and adjuvant radiotherapy is recommended for high-risk cases (9).

Given the long latent period and wide range of recurrence intervals for ectopic CP (ranging from 0.08 to 34 years, with a mean of 7.24 ± 7.86 years), long-term regular follow-up and lifelong re-examinations are required for patients postoperatively. Cranial MRI should be performed at 1, 3, and 6 months postoperatively to evaluate tumor residue or early implantation in the surgical area and tract (10). From 1 to 5 years postoperatively, routine cranial MRI, pituitary-hypothalamic endocrine function tests, visual field assessments, and neurological evaluations should be conducted annually. Beyond 5 years, annual regular re-examinations are still necessary to detect ectopic recurrent lesions along the surgical tract and at distant sites (6). Once new symptoms such as headache, visual loss, or endocrine disorders occur, patients should seek medical examination immediately to enable early detection and intervention of recurrent lesions.

This study has several limitations. First and foremost, no cyst fluid specimens were harvested for laboratory analysis during either primary tumor resection or secondary reoperation for recurrent lesions. The cyst fluid of aCP harbors numerous inflammatory mediators, viable tumor cells, and specific proteins, which can induce local inflammatory responses and promote tumor cell shedding, adhesion, and subsequent ectopic implantation (11). The lack of cytological, biochemical, and molecular profiling of intraoperative cyst fluid in both procedures precluded direct verification of viable tumor cells within the cyst fluid and quantitative assessment of how cystic components contribute to surgical tract seeding and ectopic recurrence. Furthermore, the absence of paired cyst fluid samples hindered the analysis of dynamic alterations in tumor proliferative activity and inflammatory status between the two surgeries, thereby weakening the evidentiary basis for our mechanism that intraoperative cyst fluid leakage drives ectopic tumor recurrence (12). For future studies, routine collection and systematic multimodal testing of intraoperative cyst fluid are strongly advocated to further elucidate the pathogenesis of CP ectopic recurrence and optimize intraoperative tumor-free surgical strategies.

## Conclusion

Adamantinomatous craniopharyngioma is susceptible to cyst rupture and tumor dissemination, carrying a markedly higher risk of ectopic recurrence compared with the predominantly solid and less invasive papillary subtype. Strict adherence to tumor-free surgical principles intraoperatively helps prevent tumor rupture. The surgical field and tract should be thoroughly irrigated, and gross total resection of the primary lesion should be pursued whenever possible. For high-risk tumors tightly adherent to adjacent vital neurovascular structures, subtotal resection combined with adjuvant radiotherapy is recommended to reduce the risks of tumor residue and dissemination.

This study has several limitations. Cyst fluid specimens were not collected for cytological, biochemical and molecular analyses during either operation. Accordingly, we could not directly confirm the presence of viable tumor cells in the cyst fluid, nor explore how dynamic changes in cyst fluid components contribute to tumor seeding and recurrence. Future clinical studies should attach importance to the collection and comprehensive testing of intraoperative cyst fluid samples. This will help further clarify the mechanisms underlying ectopic recurrence of craniopharyngioma and optimize surgical prevention and treatment strategies.

## Data Availability

The original contributions presented in the study are included in the article/**Supplementary Material**. Further inquiries can be directed to the corresponding author.
