# Supplementary material for: Ectopic craniopharyngioma recurrence: a case report and literature review
Source: Front Oncol. 2026 Jul 8;16:1880297. doi: 10.3389/fonc.2026.1880297 (PMC13388166; doi:10.3389/fonc.2026.1880297)
Supplement: Supplementary file 1 [file SupplementaryFile1.docx]

7. Nogueira J, Sobreiro Silva J, Marques R, Antunes C, Pereira R, Afonso Filipe M.

Ectopic craniopharyngioma recurrence: a case report and literature review. Cureus.

(2024) 16:e69607. doi: 10.7759/cureus.69607

8. Selfa A, Arraez C, Ros A, Linares J, Cerro L, Arraez M. Ectopic recurrence of

craniopharyngioma in the posterior fossa: case report and review of the literature.

Neurocirugia (Engl Ed). (2023) 34:32–9. doi: 10.1016/j.neucie.2022.11.001

10. Mahdi MA, Krauss JK, Nakamura M, Brandis A, Hong B. Early ectopic recurrence

of craniopharyngioma in the cerebellopontine angle. Turk Neurosurg. (2018) 28:313–6.

doi: 10.5137/1019-5149.jtn.17215-16.1

12. Schmalisch K, Beschorner R, Psaras T, Honegger J. Postoperative intracranial

seeding of craniopharyngiomas–report of three cases and review of the literature. Acta

Neurochir (Wien). (2010) 152:313–9. doi: 10.1007/s00701-009-0538-4

13. Marquez YB, Johnson AR, Cafferata GN, Jaimovich SG. Analysis of the different

pathways of ectopic recurrence of craniopharyngioma in pediatric patients: presenta

tion of cases and review of the literature. Childs Nerv Syst. (2024) 40:3511–8. doi:10.1007/s00381-024-06585-4

14. Steed T, Indelicato DJ, Souster J, van Landeghem FKH, Mehta V, Patel S. Spinal

ectopic recurrence of craniopharyngioma in a pediatric patient. Childs Nerv Syst. (2023)

39:279–84. doi: 10.22541/au.164872446.66300619/v1

15. Ji C, Cheng H, Zhou X, Cao X, Qiao N, Shi C, et al. Ectopic recurrence

craniopharyngioma: series report and literature review. Chin Neurosurg J. (2023)

9:13. doi: 10.1186/s41016-023-00326-3

16. Carfagno VF, Rouintan J, Ahmed I. Ectopic recurrence of a craniopharyngioma.

Cureus. (2023) 15:e35988. doi: 10.7759/cureus.35988

17. Loh A, Germann J, Qazi S, Husain R, Boutet A, Lozano AM, et al. Lesion network

mapping of ectopic craniopharyngioma identifies potential cause of psychosis: a case

report. Acta Neurochir (Wien). (2022) 164:3285–9. doi: 10.1007/s00701-022-05355-y

18. Cai M, He H, Zhang B, Luo L, Gong J, Li W, et al. An ectopic recurrent

craniopharyngioma of the temporal lobe: case report and review of the literature.

World Neurosurg. (2019) 126:216–22. doi: 10.1016/j.wneu.2019.02.196

19. Renfrow JJ, Greeneway GP, Carter L, Couture DE. Intraventricular recurrence of a

craniopharyngioma: case report. J Neurosurg Pediatr. (2018) 22:393–6. doi: 10.3171/

2018.4.peds18112

20. Jian XD, Shrestha D, Zhuang Z. Craniopharyngioma: a case report of ectopic

recurrence. Turk Neurosurg. (2017) 27:160–2. doi:10.5137/1019-5149.JTN.12386-14.2

21. Carleton-Bland N, Kilday JP, Pathmanaban ON, Stivaros S, Kelsey A, Kamaly-Asl

ID. Ventricular metastatic dissemination of a paediatric craniopharyngioma: case

report and literature review. Br J Neurosurg. (2017) 31:474–7. doi: 10.3109/

02688697.2016.1139050

22. Du C, Feng CY, Yuan J, Yuan X. Ectopic recurrence of pediatric

craniopharyngiomas after gross total resection: a report of two cases and a review of

the literature. Childs Nerv Syst. (2016) 32:1523–9. doi: 10.1007/s00381-016-3050-1

23. Yang Y, Shrestha D, Shi XE, Zhou Z, Qi X, Qian H. Ectopic recurrence of

craniopharyngioma: reporting three new cases. Br J Neurosurg. (2015) 29:295–7.

doi: 10.3109/02688697.2014.967751

24. Clark SW, Kenning TJ, Evans JJ. Recurrent ectopic craniopharyngioma in the

sylvian fissure thirty years after resection through a pterional approach: a case report

and review of the literature. Nagoya J Med Sci. (2015) 77:297–306.

25. Goncalves CB, Lima GA, Nogueira J, do Souto AA, Chimelli L, Taboada GF.

Subgaleal recurrence of craniopharyngioma of rapid growing pattern. Pituitary. (2014)

17:214–9. doi:10.1007/s11102-013-0490-4

26. Jakobs M, Orakcioglu B. Ectopic recurrent craniopharyngioma of the frontal bone.

J Neurosurg. (2012) 117:490–7. doi: 10.3171/2012.6.jns111885

27. Roldan-Serrano MA, Katati MJ, Garcıa-Lopez C, Iañez-Velasco B, Sanchez-Corral

C, Altuzarra-Corral A, et al. Ectopic relapse of an operated craniopharyngioma. Case

report and review of the literature. Neurocirugia (Astur). (2011) 22:439–44.

28. Salunke P, Bhardwaj LK, Gupta K, Das K. Ectopic recurrence of

craniopharyngioma along the surgical tract. Neurol India. (2011) 59:137–9.

doi: 10.4103/0028-3886.76881

29. Kordes U, Flitsch J, Hagel C, Goebell E, Schwarz R, Herberhold T, et al. Ectopic

craniopharyngioma. Klin Padiatr. (2011) 223:176–7. doi: 10.1055/s-0031-1273743

30. Elfving M, Lundgren J, Englund E, Strömblad LG, Erfurth EM. Ectopic recurrence

of a craniopharyngioma in a 15-year-old girl 9 years after surgery and conventional

radiotherapy: case report. Childs Nerv Syst. (2011) 27:845–51. doi: 10.1007/s00381-010-

1375-8

31. de Blank PM, Minturn JE. A rare case of ectopic recurrence of a

craniopharyngioma diagnosed 17 years after initial presentation. J Pediatr Hematol

Oncol. (2011) 33:392–7. doi: 10.1097/mph.0b013e31820acfb2

32. Wang XY, Xu SJ, Li XG. Post-operative implantation metastasis of

craniopharyngioma: a case report. J Int Med Res. (2010) 38:1876–82. doi: 10.1177/

147323001003800536

33. Lermen O, Frank S, Hassler W. Postoperative spinal recurrence of

craniopharyngioma. Acta Neurochir (Wien). (2010) 152:309–11. doi: 10.1007/s00701-

009-0537-5

34. Romani R, Niemelä M, Celik O, Isarakul P, Paetau A, Hernesniemi J. Ectopic

recurrence of craniopharyngioma along the surgical route: case report and literature

review. Acta Neurochir (Wien). (2010) 152:297–302. doi: 10.1007/s00701-009-0415-1

35. Elliott RE, Moshel YA, Wisoff JH. Surgical treatment of ectopic recurrence of

- craniopharyngioma. Report of 4 cases. J Neurosurg Pediatr. (2009) 4:105–12. doi: [10.3171/2009.3.PEDS0948](https://doi.org/10.3171/2009.3.peds0948)

36. Frangou EM, Tynan JR, Robinson CA, Ogieglo LM, Vitali AM. Metastatic

craniopharyngioma: case report and literature review. Childs Nerv Syst. (2009)

25:1143–7. doi: 10.1007/s00381-009-0917-4

37. Bikmaz K, Guerrero CA, Dammers R, Krisht AF, Husain MM. Ectopic recurrence

of craniopharyngiomas: case report. Neurosurgery. (2009) 64:E382–3. doi:10.1227/01.NEU.0000337078.60998.98

38. Novak Z, Chrastina J, Feitova V, Lzicarova E, Rıha I. Minimally invasive treatment

of posterior fossa craniopharyngioma by means of navigated endoscopy. Minim

Invasive Neurosurg. (2008) 51:165–8. doi:10.1055/s-2008-1062743

39. Jeong IH, Lee JK, Moon KS, Joo SP, Kwak HJ, Kim TS, et al. Ectopic recurrence of

craniopharyngioma: a case report and review of the literature. J Neuro-Oncol. (2006)

79:191–5. doi: 10.1007/s11060-006-9124-2

40. Yamada Y, Haraoka J, Akimoto J. Ectopic recurrence of craniopharyngioma.

Neurol Med Chir (Tokyo). (2006) 46:598–600. doi:10.2176/nmc.46.598

41. Bianco Ade M, Madeira LV, Rosemberg S, Shibata MK. Cortical seeding of a

craniopharyngioma after craniotomy: case report. Surg Neurol. (2006) 66:437–40.

doi: 10.1016/j.surneu.2005.12.027

42. Kawaguchi T, Fujimura M, Shirane R, Shoji T, Watanabe M, Tominaga T. Ectopic

recurrence of craniopharyngioma. J Clin Neurosci. (2005) 12:307–9. doi: 10.1016/

j.jocn.2004.01.009

43. Ishii K, Sugita K, Kobayashi H, Kamida T, Fujiki M, Izumi T, et al. Intracranial

ectopic recurrence of craniopharyngioma after Ommaya reservoir implantation.

Pediatr Neurosurg. (2004) 40:230–3. doi: 10.1159/000082297

44. Novegno F, Di Rocco F, Colosimo C, Lauriola L, Caldarelli M. Ectopic recurrences

of craniopharyngioma. Childs Nerv Syst. (2002) 18:468–73. doi: 10.1007/s00381-002-

0641-9

45. Fuentes S, Metellus P, Dufour H, Do L, Grisoli F. Postoperative intracranial seeding

of craniopharyngioma. Three case reports and a review of the literature.

Neurochirurgie. (2002) 48:345–50.

46. Elmaci L, Kurtkaya-Yapicier O, Ekinci G, Sav A, Pamir MN, Vidal S, et al.

Metastatic papillary craniopharyngioma: case study and study of tumor angiogenesis.

Neuro Oncol. (2002) 4:123–8. doi: 10.1215/15228517-4-2-123

47. Liu JM, Garonzik IM, Eberhart CG, Sampath P, Brem H. Ectopic recurrence of

craniopharyngioma after an interhemispheric transcallosal approach: case report.

Neurosurgery. (2002) 50:639–44. doi: 10.1227/00006123-200203000-00041

48. Kim SK, Wang KC, Shin SH, Choe G, Chi JG, Cho BK. Radical excision of pediatric

craniopharyngioma: recurrence pattern and prognostic factors. Childs Nerv Syst. (2001)

17:531–7. doi: 10.1007/s003810100458

49. Lee DK, Jung HW, Kim DG, Paek SH, Gwak HS, Choe G. Postoperative spinal

seeding of craniopharyngioma. Case report. J Neurosurg. (2001) 94:617–20. doi:10.3171/jns.2001.94.4.0617

50. Ito M, Jamshidi J, Yamanaka K. Does craniopharyngioma metastasize? Case report

and review of the literature. Neurosurgery. (2001) 48:933–6. doi: 10.1097/00006123-

200104000-00050

51. Freitag SK, Miller NR, Brem H. Recurrent ectopic craniopharyngioma. Br J

Neurosurg. (2001) 15:511–3. doi: 10.1080/02688690120097723

52. Lee JH, Kim CY, Kim DG, Jung HW. Postoperative ectopic seeding of

craniopharyngioma. Case illustration. J Neurosurg. (1999) 90:796. doi:10.3171/jns.1999.90.4.0796

53. Gupta K, Kuhn MJ, Shevlin DW, Wacaser LE. Metastatic craniopharyngioma.

AJNR Am J Neuroradiol. (1999) 20:1059–60. doi: 10.1016/b978-0-7216-8148-1.50098-x

54. Israel ZH, Pomeranz S. Intracranial craniopharyngioma seeding following radical

resection. Pediatr Neurosurg. (1995) 22:210–3. doi: 10.1159/000120903

55. Keohane C, Hally M, Ryder DQ, Buckley TF. Late recurrence of

craniopharyngioma in the cerebellopontine angle in a fertile woman. J Neurol

Neurosurg Psychiatry. (1994) 57:873–4. doi: 10.1136/jnnp.57.7.873

56. Tomita T, McLone DG. Radical resections of childhood craniopharyngiomas.

Pediatr Neurosurg. (1993) 19:6–14. doi: 10.1159/000120693

57. Malik JM, Cosgrove GR, VandenBerg SR. Remote recurrence of

craniopharyngioma in the epidural space. Case report. J Neurosurg. (1992) 77:804–7.

doi: 10.3171/jns.1992.77.5.0804

58. Tomita S, Mendoza ND, Symon L. Recurrent craniopharyngioma in the posterior

fossa. Br J Neurosurg. (1992) 6:587–90. doi: 10.3109/02688699209002377

59. Ragoowansi AT, Piepgras DG. Postoperative ectopic craniopharyngioma. Case

report. J Neurosurg. (1991) 74:653–5. doi:10.3171/jns.1991.74.4.0653

60. Gökalp HZ, Egemen N, Ildan F, Bacaci K. Craniopharyngioma of the posterior

fossa. Neurosurgery. (1991) 29:446–8. doi: 10.1097/00006123-199109000-00020

61. Barloon TJ, Yuh WT, Sato Y, Sickels WJ. Frontal lobe implantation of

craniopharyngioma by repeated needle aspirations. AJNR Am J Neuroradiol. (1988)

9:406–7.
