# Supplementary material for: Ectopic craniopharyngioma recurrence: a case report and literature review
Source: Front Oncol. 2026 Jul 8;16:1880297. doi: 10.3389/fonc.2026.1880297 (PMC13388166; doi:10.3389/fonc.2026.1880297)
Supplement: Supplementary file 2 [file Table1.doc]

| Author, year | Age/Sex | Ectopic recurrence location | Ectopic recurrence mechanism | Interval between the firstsurgery and ectopicrecurrence | Symptoms upon first discovery | Symptoms of recurrence | Previous treatments | Histology |
| --- | --- | --- | --- | --- | --- | --- | --- | --- |
| Present | 67/M | Right temporal lobe | Surgical tract implantation | 4 years | Blurred vision | Headache | Cranio GTR (first); cranio  GTR (second); | Adamantinomatous |
| Nogueira, J. et al,2024 | 77/M | Left frontal lobe | CSF | 4 years | Progressive visual loss | Bilateral visual deficit with an almost amaurotic right eye | Cranio GTR (first); cranio  STR (second); cranio  STR+RT (third) | Adamantinomatous |
| Márquez, Y. B. et al,2024 | 6/M | Temporal intraparenchymal | Subarachnoid dissemination | 3 years | progressive headache and vomiting | NM | Cranio STR (first); cranio  GTR (second); | Adamantinomatous |
|  | 9/F | Intraosseous orbital | Surgical tract implantation | 2 years | NM | right eyelid ptosis | Cranio GTR (first); cranio  GTR (second); | Adamantinomatous |
|  | 4/M | Occipital intraventricular | CSF | 8 years(first),2 years(second) | progressive headache and vomiting | intracranial hyper-  tension | Cranio GTR (first); cranio  GTR (second); cranio  GTR(third) | Adamantinomatous |
| Steed, T. et al, 2023 | 10/F | Spine | CSF | 5 years | Left eye diplopia, left eyelid droop | Upper back pain | Cranio STR (first); cranio  GTR (second); | Adamantinomatous |
| Selfa, A. et al,2023 | 7/M | CPA | CSF | 20 years | NM | NM | Cranio STR (first); cranio  GTR (second); | Adamantinomatous |
|  | 36/M | Right cerebellar hemisphere | CSF | 18 years | NM | Gait disorders, mental retardation | NM | Adamantinomatous |
| Ji, C. et al,2023 | 49/F | Right frontal lobe | Surgical tract implantation | 7 years(First),9 years(Second) | Vision loss, nausea | Severe nausea, vomiting | Cranio GTR (first); cranio  GTR (second) | Adamantinomatous |
|  | 63/F | Right temporal lobe | CSF | 2 years | Bilateral vision loss | Recurrent vision loss; aggravated symptoms | Cranio STR with Ommaya catheter implantation | Adamantinomatous |
| Carfagno, V. F. et al,2023 | 27/M | Splenius of the corpus callosum | CSF | 10 years | NM | Persistent headache | NM | NM |
| Loh, A. et al,2022 | 60/M | Right temporal lobe | CSF | 10 years | NM | Hallucinations, paranoid delusions, inappropriate laughter | Cranio GTR (first); cranio  GTR (second) | Adamantinomatous |
| Cai, M. et al,2019 | 28/M | Right temporal lobe | CSF | 1 year | Progressive headache | Headache | Cranio GTR (first); cranio  GTR (second) | Adamantinomatous |
| Renfrow, J. J. et al,2018 | 14/F | left lateral ventricle | CSF | 2 years | Ataxia, irritability, vomiting, and obstructive hydrocephalus | Worsening headaches | Cranio GTR (first); cranio  GTR (second);RT(Third) | Adamantinomatous |
| Mahdi, M. A. et al,2018 | 24/M | Right CPA | CSF | 4 months | NM | NM | Cranio GTR (first); cranio  GTR (second) | Adamantinomatous |
| Jian, X. D. et al,2017 | 42/M | Right frontal lobe | Surgical tract implantation | 5 years | Headache and decreased visual acuity | Severe headache and dizziness | Cranio GTR (first); cranio  GTR (second) | Papillary |
| Carleton-Bland , N. et al, 2017 | 4/M | Right lateral ventricle | CSF | 6 years | Vomiting, headaches, lethargy | NM | Cranio STR (first); cranio  GTR+RT | Adamantinomatous |
| Du, C. et al,2016 | 6/F | Right frontal lobe | Surgical tract implantation | 5 years | Growth retardation, progressive headache, vomiting | Peritumoral edema | Cranio GTR (first); cranio  GTR (second) | Adamantinomatous |
|  | 4/M | 4th ventricular | CSF | 4 years | Polydipsia, polyuria, progressive headache, and vomiting | Rapid visual deterioration | Cranio GTR (first); cranio  GTR (second) | Adamantinomatous |
| Yang, Y. et al,2015 | 44/M | Right frontal lobe | Surgical tract implantation | 9 years | NM | Tonic-clonic seizure | Cranio GTR (first); cranio  GTR (second) | Papillary |
|  | 46/M | Interhemispheric cistern | Surgical tract implantation | 2 years | Decreased libido and hypodynamia | Headache and vomiting | Cranio GTR (first); cranio  GTR (second) | Papillary |
|  | 48/M | Right frontotemporal lobe | Surgical tract implantation | 6 years | NM | eft headache, visual loss | Cranio GTR (first); cranio  GTR (second) | Adamantinomatous |
| Clark, S. W. et al,2015 | 35/M | Right frontal lobe | Surgical tract implantation | 9 years | NM | NM | Cranio GTR (first); cranio  GTR (second) | NM |
|  | 33/F | Left temporal sylvian cistern | Surgical tract implantation | 34 years | Bitemporal hemianopsia | NM | Cranio GTR (first); cranio  GTR (second) | Adamantinomatous |
| Gonçalves, C. B. et al,2014 | 49/M | Right frontal subgaleal space | Surgical tract implantation | 5 years | Headache and visual field defect | A rapidly growing lump at the right frontal surgical incision site | Cranio GTR (first); cranio  GTR (second) | Adamantinomatous |
| Jakobs, M. et al,2012 | 61/F | Right supraorbital frontal bone | Surgical tract implantation | 16 years | headache, diplopia, and new-onset bitemporal hemianopia | Painless swelling above the right eye | Cranio GTR (first); RT(second) | Adamantinomatous |
| Roldán-Serrano, M. A. et al,2011 | 69/F | Right frontal lobe | Surgical tract implantation | 10 years | NM | Transient mandibular locking episodes | Cranio GTR (first); cranio  GTR (second) | Adamantinomatous |
| Salunke, P. te al, 2011 | 5/F | Right Sylvian fissure | Surgical tract implantation | 11 months | Left limb weakness, progressive loss of right vision | NM | Cranio STR (first); cranio  GTR (second) | Adamantinomatous |
| Kordes, U . et al, 2011 | 7/M | Right pterional lobe | Surgical tract implantation | 15 months | Intermittent headache, fatigue | NM | Cranio STR (first); cranio  GTR (second | Adamantinomatous |
| Elfving, M. et al,2011 | 4/F | Right frontal lobe | Surgical tract implantation | 11 years | Central TSH and ACTH insufficiency | NM | Cranio GTR (first); cranio  GTR (second) | Adamantinomatous |
| Minturn, J. E. et al, 2011 | 5/F | Left CPA | CSF | 17 years | NM | Intermittent headache and ataxia | Cranio GTR (first); cranio  GTR (second) | Adamantinomatous |
| Wang, X. Y. et al, 2010 | 3/M | Subdural space of the right frontal lobe | Surgical tract implantation | 2 years | Progressive headache and bitemporal hemianopsia | Headache, dizziness and generalized tonic-clonic seizures | Cranio GTR (first); cranio  GTR (second) | Adamantinomatous |
| Schmalisch, K. et al, 2010 | 13/M | Right sylvian fissure | Surgical tract implantation | 2 years | NM | NM | Cranio GTR (first); cranio  GTR (second) | Adamantinomatous |
|  | 27/F | Right frontolateral region | Surgical tract implantation | 2 years | NM | NM | Cranio GTR (first); cranio  GTR (second) | Adamantinomatous |
|  | 42/M | Right parietal lobe | CSF | 10 years | NM | NM | Cranio GTR (first); cranio  GTR (second) | Adamantinomatous |
| Lermen, O. et al,2010 | 49/M | Lumbar space | CSF | 4 years(First)，6 months(Second) | Sudden visual impairment | Progressive low-back pain | Cranio GTR (first); cranio  GTR (second) | Adamantinomatous |
| Romani, R. et al, 2010 | 18/F | Right medial frontal lobe | Surgical tract implantation | 4 years | Progressive visual field deficit | NM | Cranio GTR (first); cranio  GTR (second) | Adamantinomatous |
| Elliott, R. E. et al, 2009 | 3/F | Right prepontine cistern | CSF | 10 years | Progressive headache, left homonymous hemianopia | NM | Cranio GTR (first); cranio  GTR (second) | Adamantinomatous |
|  | 2/M | Left CPA | CSF | 4 years(First),21 months(Second) | Headache, bitemporal hemianopia | NM | Cranio GTR (first); cranio  GTR (second);cranio  GTR (third) | Adamantinomatous |
|  | 3/F | Ipsilateral right sylvian fissure, underneath the right optic nerve | Surgical tract implantation | 20 months | Progressive headache | NM | Cranio GTR (first); cranio  GTR (second) | Adamantinomatous |
|  | 6/M | Contralateral subfrontal region | Uncertain | 1 month | Severe headache, visual impairment | NM | Cranio GTR (first); cranio  GTR (second) | Adamantinomatous |
|  | 37/F | Prepontine | CSF | 12 years | NM | NM | NM | NM |
| Frangou, E. M. et al, 2009 | 10/M | Right parietal lobe | CSF | 4 years | Decreased visual acuity | NM | Cranio STR (first); cranio  STR (second); cranio  STR+RT (third) | Adamantinomatous |
| Bikmaz, K. et al,2009 | 52/F | Prepontine cistern | CSF | 15 years | NM | Balance disturbance, diplopia | Cranio GTR (first); cranio  STR (second); | Adamantinomatous |
|  | 41/M | Right frontal lobe | Surgical tract implantation | 9 years | Headache, visual disturbance | Headache, dizziness, disequilibrium, right upper extremity drift | Cranio GTR (first); cranio  GTR (second) | Papillary |
|  | 24/M | Bilateral CPA | CSF | 12 years | NM | Right facial numbness, drowsiness | Cranio STR+RT (first); cranio  GTR +gamma knife(second) | NM |
| Novák, Z. et al,2008 | 48/M | Posterior fossa | CSF | 19 years | Optic nerve compression caused by primary suprasellar CP | Symptoms of cerebellar and brainstem compression, nocturnal confusion | Cranio GTR (first); cranio  GTR (second);cranio  GTR (third) | NM |
| Jeong, I. H. et al, 2006 | 8/F | Right frontal lobe | Surgical tract implantation | 4 years | Decreased visual acuity, progressive headache | Generalized tonic-clonic seizure | Cranio GTR (first); cranio  GTR (second) | Adamantinomatous |
| Yamada, Y. et al,2006 | 17/M | Left frontal lobe | CSF | 10 years | Amenorrhea, right temporal hemianopsia, minor right visual disturbance | NM | Cranio STR (first); cranio  GTR (second) | Adamantinomatous |
| Bianco Ade, M. et al,2006 | 27/M | Left temporal lobe | Surgical tract implantation | 1 year | Progressive visual loss | Refractory seizures, hallucinations, psychotic episodes | Cranio GTR (first); cranio  GTR (second) | Adamantinomatous |
| Kawaguchi, T. et al,2005 | 50/M | Left frontal lobe | Surgical tract implantation | 2 years | Right visual acuity disturbance | NM | Cranio GTR (first); cranio  GTR (second) | Adamantinomatous |
| Ishii, K. rt al,2004 | 2/M | Subdural space in the center of the frontal base under the craniotomy bone flap, surrounding the Ommaya reservoir cannula | Surgical tract implantation | 4 months,10 months | Headache, nausea, lethargy, accompanied by acute hydrocephalus | NM | Cranio GTR (first); cranio  GTR (second);cranio  GTR (third) | Adamantinomatous |
| Novegno, F. et al,2002 | 6/M | Left fronto-basal convexity cortex(First); Left ventral pons, right anterior lentiform nucleus, floor of the third ventricle near mammillary bodies(Second) | Surgical tract implantation(First);CSF(Second) | 3 years(First),1 year(Second) | Diplopia, progressive worsening headaches, reduced visual acuity, bilateral papilledema, slight paresis of both abducens nerves | NM | Cranio STR (first); cranio  GTR (second);No surgery (third) | Adamantinomatous |
| Fuentes, S. et al,2001 | 32/M | Right frontal lobe | Surgical tract implantation | 5 years | NM | NM | Cranio GTR (first); cranio  GTR (second) | Adamantinomatous |
|  | 11/M | NM | Surgical tract implantation | 3 years | NM | NM | Cranio GTR (first); cranio  GTR (second) | Adamantinomatous |
|  | 9/M | Right frontal lobe; right  temporal lobe | Surgical tract implantation | 10 years | NM | NM | Cranio GTR (first); cranio  GTR (second) | Adamantinomatous |
| Elmaci , L. et al,2001 | 62/F | Left temporal lobe | CSF | 2 years | Headache, dizziness, visual disturbance | Headache unresponsive to analgesics | Cranio GTR (first); cranio  GTR (second) | Papillary |
| Liu, J. M. et al,2002 | 73/M | Right frontal lobe | Surgical tract implantation | 8 years | Progressive headaches, dizziness, loss of short-term memory with hydrocephalus | Decline in neurocognitive status, left lower extremity weakness, dizziness, generalized fatigue, intermittent headaches | Cranio GTR (first); cranio  GTR (second);cranio  GTR | Adamantinomatous |
| Kim, S. K. et al, 2001 | Child,NM | Internal auditory canal | CSF | NM | NM | NM | NM | NM |
| Lee, D. K. et al,2001 | 26/M | Intradural extramedullary lesions in the lumbosacral spinal canal | CSF | 19 months | Headache, decreased visual acuity in both eyes | Low-back pain, right radiating leg pain | Cranio STR (first); cranio  STR (second) | Papillary |
| Ito, M. et al,2001 | 65/M | Right temporal lobe | CSF | 3 years | Persistent visual field defect | NM | Cranio GTR (first); cranio  GTR (second) | Adamantinomatous |
| Freitag, S. K. et al,2001 | 66/F | Right frontal lobe | Surgical tract implantation | 8 years | Progressive headache, dizziness, loss of short-term memory with hydrocephalus and bitemporal visual field defects | NM | Cranio GTR (first); cranio  GTR (second) | NM |
| Lee, J. H. et al,1999 | 31/M | Right frontal lobe | Surgical tract implantation | 2 years | NM | NM | NM | NM |
| Gupta, K. et al, 1999 | 73/M | Left parietal lobe and  left frontal lobe | CSF | 7 years | Bitemporal hemianopsia | New onset of partial seizures | Cranio GTR (first); cranio  GTR (second | Adamantinomatous |
| Israel, Z. H. et al,1995 | 12/M | Right frontal lobe | Surgical tract implantation | 2 years | NM | Severe headache, vomiting, diplopia | Cranio GTR (first); cranio  GTR (second) | NM |
| Keohane, C. et al,1994 | 7/F | Left CPA | CSF | 26 years | NM | Left facial numbness and paresthesia | Cranio STR (first); cranio  STR (second) | Adamantinomatous |
| Tomita, T. et al,1993 | Child/NM | Right frontal lobe | Surgical tract implantation | NM | Headache, visual disturbance, growth retardation | NM | Cranio STR (first); cranio  GTR (second) | Adamantinomatous |
| Malik, J. M. et al,1992 | 6/M | Epidural space of the right frontal area | Surgical tract implantation | 21 years | Headaches, falls, personality change | Intermittent frontal and occipital headaches | Cranio GTR (first); cranio  GTR (second) | Adamantinomatous |
| Tomita, S. et al,1992 | 48/F | Right CPA, | CSF | 25 years | Foster-Kennedy syndrome | Headache, loss of balance, difficulty walking | Cranio GTR (first); cranio  GTR (second) | Adamantinomatous |
| Ragoowansi, A. T. et al,1991 | 47/M | Right sylvian fissure | Surgical tract implantation | 2 years | Decreased visual acuity, retro-orbital headaches, progressive fatigue, decreased libido | NM | Cranio GTR (first); cranio  GTR (second) | NM |
| Gökalp, H. Z. et al,1991 | 3/M | 4th ventricle | CSF | 20 years | Headache, loss of vision | Headache, vomiting, nausea, gait disturbance | Cranio GTR (first); cranio  GTR (second) | Adamantinomatous |
| Barloon, T. J. et al,1988 | 5/M | Right frontal lobe | Surgical tract implantation | 10 years | NM | NM | Cranio STR RT (first);  cyst aspiration+RT(Second) | Adamantinomatous |
